# Supplementary material for: Optimization and CFD-RSM analysis of single orifice reactor for enhanced biodiesel production
Source: PLoS One. 2025 Jul 2;20(7):e0326747. doi: 10.1371/journal.pone.0326747 (PMC12221185; doi:10.1371/journal.pone.0326747)
Supplement: Attachment — Submitted filename: Manuscript_Review.docx [file pone.0326747.s002.docx]

# Manuscript Review: Optimization and CFD-RSM Analysis of Single Orifice Reactor for Enhanced Biodiesel Production

## 1. Key Scientific Contributions and Strengths

This study presents a well-structured approach to optimizing a single-orifice oscillatory flow reactor (OFR) for biodiesel production, utilizing Computational Fluid Dynamics (CFD) and Response Surface Methodology (RSM). The combination of fluid dynamic modeling and experimental validation is a strong aspect, particularly the attempt to correlate Turbulent Kinetic Energy (TKE), vorticity, and biodiesel yield. One of the major highlights is that the optimized reactor conditions (12.12 Hz frequency, d₀/D = 0.4-, and 10 mm baffle spacing) led to an 83% biodiesel conversion, showing the potential of oscillatory reactors for efficient mixing and mass transfer. The methodology, which involves varying baffle diameter ratio, frequency, and spacing, then using RSM to derive optimal conditions, is sound. By sharing their observation on the effects of pressure drop, vorticity, and turbulence intensity, the study contributes to efficient design of reactors for biodiesel yield.

## 2. Revisions/clarifications required

### 2.1. Lack of Clear Justification for Optimal Conditions

The manuscript states that the optimized reactor parameters were a frequency of 12.12 Hz, a baffle diameter ratio (d₀/D) of 0.4, and a baffle spacing of 10 mm (Abstract). However, it does not clearly explain why these conditions were chosen over others within the tested range. In Table 2, the study varied d₀/D between 0.4 - 0.8, frequency between 5 - 15 Hz, and baffle spacing between 3 - 7 mm. But the manuscript does not explicitly state how biodiesel yield varied with these parameters, making it unclear whether these values were chosen purely based on TKE, vorticity, or a trade-off with biodiesel yield.

### 2.2. Insufficient Discussion on Practical Limitations and Industrial Feasibility

The study convincingly shows that higher frequency and increased turbulence enhance biodiesel yield, but it does not discuss real-world operational limitations of oscillatory flow reactors. High-frequency oscillations may improve mixing, but they also increase energy consumption. There is no mention of whether the energy cost associated with high oscillation frequencies offsets the gain in biodiesel yield.

### 2.3. CFD Model Limitations and Assumptions

The manuscript models the reactor using a single-phase flow assumption with the k-ε turbulence model (Section 2.3.1). While k-ε is widely used, it struggles with near-wall turbulence predictions and may underpredict the effects of flow separation around baffles. The authors should briefly explain why they did not use a k-ω SST model which would provide a more accurate representation of turbulence and vortex shedding.

### 2.4. Experimental Validation Lacks GC Details

The experimental validation section (2.5) states that biodiesel yield was measured using Gas Chromatography (GC), but does not provide details such as: Gc sample preparation? the general settings of the GC? Specifications of the GC used? This will help with the reproducibility and comparison of results.

### 3. Declaration

I could not correlate the figure discussions to figures as the figures were not attached to the manuscript.

## 4. Recommendation

This manuscript presents a valuable contribution to the field of biodiesel reactor optimization and should be accepted for publication after the minor revisions are concisely done.
