# Supplementary material for: Optimization and CFD-RSM analysis of single orifice reactor for enhanced biodiesel production
Source: PLoS One. 2025 Jul 2;20(7):e0326747. doi: 10.1371/journal.pone.0326747 (PMC12221185; doi:10.1371/journal.pone.0326747)
Supplement: Attachment — Submitted filename: PLOS ONE comments.docx [file pone.0326747.s003.docx]

**Paper Title**: Optimization and CFD-RSM Analysis of Single Orifice Reactor for Enhanced Biodiesel Production

**Journal Title:** PLOS ONE

**Manuscript No.:** PONE-D-25-06866

**Article type:** Research Article

In the present investigation titled “Optimization and CFD-RSM Analysis of Single Orifice Reactor for Enhanced Biodiesel Production” demonstrates the potential of single orifice reactor to enhance biodiesel production through improved turbulance and mixing efficiency. Further the optimal dimensions of the reactor design were determined using numerical simulations and experimental analysis. role of O-H bond scission in ethanol steam reforming is emphasized. The topic is relevant given the increasing global interest in sustainable energy solutions and the pivotal role that biodiesel is expected to play in the transition towards a clean energy future. However, there are a few areas where the manuscript could be further improved with minor revisions:

1. Design the Abstract part and it must be more informative by including more mathematical findings and more powerful explanations (For e.g., also discuss the parameters effect on biodiesel yield). Please be concise-maximum 1-2 lines.
2. The author should use new and more references in the introduction (2024, 2023, 2022 , …).
3. For consistency, write the abbreviation of the terminology and used these abbreviations throughout the manuscript and avoid repetition of terminologies [e.g., turbulent kinetic energy (TKE), computational fluid dynamics (CFD), etc…].
4. In between the words, the capital words have been used. Please revise these.
5. In result discussion section, the authors discuss about the result, but mentioned few outdated references. Please, discuss your results with previous recent studies.
6. Can you explain on the statistical procedures used to ensure reliable and accurate findings in the RSM modelling?
7. Citations are included but could be better integrated into the narrative. Some sections might benefit from additional references to support claims or provide further reading.
8. Author should consider adding references from the past few years (2020 onward) to reflect the latest developments in the field.
9. In Table 1, author please check the values of “the ratio of baffle diameter to reactor diameter” (For e.g., it is 0.4-0.8 or 0/4-0/8)?
10. Please check all the references cited in the text carefully and correct the inconsistency (For e.g., Line 554, check the reference format i.e., Sreenivasan & Vedantam, 2024).
11. Professional proofread should be conducted again for the whole manuscript due to many typos.
