# Supplementary material for: Optimization and CFD-RSM analysis of single orifice reactor for enhanced biodiesel production
Source: PLoS One. 2025 Jul 2;20(7):e0326747. doi: 10.1371/journal.pone.0326747 (PMC12221185; doi:10.1371/journal.pone.0326747)
Supplement: Attachment — Submitted filename: answer to reviewers.docx [file pone.0326747.s006.docx]

Response to the honorable referee of the paper entitled:

**Optimization and CFD-RSM Analysis of Single Orifice Reactor for Enhanced Biodiesel Production**

Thanking the comments and proposed amendments of the honorable referees, the answer to the referred points is mentioned in the separation of different parts of the paper as follows:

Reviewer #1

1. Better connect your research findings to previous works published in Plos One and in other top journals.

Response: Thanks for your attention. It was done.

2. Please remove ANY lumped references. Define each of them separately to avoid inappropriate citations.

Response: Thanks for your attention. It was corrected in the text.

3. It is recommended that the authors work with a science editor who is proficient in the native English language to improve the organization and delivery of some portions of the manuscript.

Response: Thanks for your recommendation. It was done.

4. Too many abbreviations are used in the analysis and results. I recommend a nomenclature section for the abbreviations and variables used throughout the passage.

Response: Thanks for mentioning that. It was prepared and added between the abstract and introduction sections.

5. The journal's author guidelines and instructions should be followed in preparing the revised version.

Response: Thanks for your comment. The manuscript was revised with plos one's author guidelines.

6. The abstract should state briefly the purpose of the research, the principal results, and major conclusions. In the abstract, please add an indication of the achievements from your study that are relevant to the journal's scope. Please be concise—maximum 1-2 lines.

Response: Thanks for mentioning that. The revised abstract now includes purpose, results, and conclusions with a brief note on practical sustainability benefits.

7. The review of the literature needs more updating with works to have a clear and concise state-of-the-art analysis. This should more clearly show the knowledge gaps identified and link them to the paper's goals.

Response: Thanks for your suggestion. The literature review in introduction section was updated with recent studies.

8. The major defect of this study is the debate or argument is not clearly stated.

Response: Thanks for mentioning that. As it was added to the end of the introduction section, the aim of this study was to design and simulate a single-orifice oscillatory reactor and analyze the turbulent kinetic energy, vorticity and dissipation factors inside this reactor to increase mixing and also increase the yield of biodiesel. In this study, the optimal reactor design dimensions were determined using numerical simulation and experimental analysis.

9. Please eliminate the use of redundant words. Eg. In this way, Recently, Respectively, therefore, currently, thus, hence, finally, to do this, first, in order, however, moreover, nowadays, today, consequently, in addition, additionally, furthermore. Please revise all similar cases, as removing these term(s) would not significantly affect the meaning of the sentence. This will keep the manuscript as CONCISE as possible. Please check ALL.

Response: Thanks for your suggestion. It was corrected in the text.

10. Avoid beginning or ending a sentence with one or a few words; they are usually redundant. Kindly revise all.

Response: Thanks for your suggestion. It was revised.

11. Some sections could benefit from clearer explanations, especially the methodology. Please avoid having one heading after another with no discussion in between, as in the case of Sections 2 and 2.1. Kindly inspect the entire document for similar instances and revise accordingly.

Response: Thanks for your suggestion. A brief explanation was added between sections 2 and 2.1.

12. Please add in the beginning your scientific hypothesis.

Response: Thanks for your suggestion. It was added at the end of the introduction section.

13. In the course of describing the performed actions, please provide reader guidance, sufficient for understanding why those actions have been performed.

Response: Thanks for your benefit comment. it was added at the beginning of each subsection in the materials and methods section. And also, it was added in flowchart (figure 1).

14. The percentage purity and company of all reagents/chemicals utilized must be reported.

Response: Thanks for your attention. It was added in section (2.5).

15. Provide more detail on the CFD parameters used. For instance, what turbulence model was applied, and why was it chosen?

Response: Thanks for mentioning that. It was added in section (2.3.1).

16. Clarify the selection and optimization process for the reactor parameters. A flowchart might help enhance understanding.

Response: Thanks for your suggestion. A flowchart (figure 1) was added to the beginning of the materials and methods section.

17. All the findings of the current work need to be compared and discussed with the results of other researchers finding instead of having a general comparison with other researchers' works. The authors should perform a comparison between the forecasting results.

Response: Thanks for your suggestion. It was added in the result and discussion section.

18. In your discussion section, please link your empirical results with a broader and deeper literature review.

Response: Thanks for your suggestion. It was added in the result and discussion section.

19. The figures and tables are adequately presented, but some graphs lack adequate labeling or legends, making it difficult to interpret the data fully.

Response: Thanks for your attention. It was corrected in the manuscript.

20. While the authors mention experimental validation of their simulations, further details on the experimental setup, conditions, and results would strengthen the manuscript.

Response: Thanks for your suggestion. It was corrected in the text

21. The discussion provides a good overview of findings, but it could be enhanced by comparing results with previous studies in the field. This would contextualize the significance of the findings and demonstrate their contribution to the field.

Response: Thanks for your suggestion. It was added in the result and discussion section.

22. Please make sure your conclusions section underscores the scientific value-added of your paper, and/or the applicability of your findings/results.

Response: Thanks, it was revised.

23. Highlights the novelty of your study in conclusion section.

Response: Thanks, it was done.

24. In the conclusions, in addition to summarizing the actions taken and results, please strengthen the explanation of their significance. It is recommended to use quantitative reasoning compared with appropriate benchmarks, especially those stemming from previous work.

Response: Thanks for your benefit comment. The conclusion was revised.

25. Ensure all cited works are up to date and relevant. Some references are older; consider including more recent studies to enhance the literature review.

Response: Thanks, it was done.

Reviewer #2

1. The manuscript states that the optimized reactor parameters were a frequency of 12.12 Hz, a baffle diameter ratio (d₀/D) of 0.4, and a baffle spacing of 10 mm (Abstract). However, it does not clearly explain why these conditions were chosen over others within the tested range. In Table 2, the study varied d₀/D between 0.4 - 0.8, frequency between 5 - 15 Hz, and baffle spacing between 3 - 7 mm. But the manuscript does not explicitly state how biodiesel yield varied with these parameters, making it unclear whether these values were chosen purely based on TKE, vorticity, or a trade-off with biodiesel yield.

Response: Thanks for your comment. A flowchart was added to section (2) to clarify the process. And also, some explanations were added to section (3.8). as it shown ranges for d₀/D, frequency, and baffle spacing (Table 2) were derived from Section (2.2) equations, then used in RSM to generate different options for reactor designs. These were simulated using CFD methods to obtain TKE, dissipations, and vorticity, which RSM analyzed to find the optimal point. Experiments validated this with an 83% biodiesel yield, linked to improved mixing. Among the tested ranges, these conditions outperformed others by balancing high TKE and vorticity with lower energy dissipation leading to the highest biodiesel yield.

2. The study convincingly shows that higher frequency and increased turbulence enhance biodiesel yield, but it does not discuss real-world operational limitations of oscillatory flow reactors.
   High-frequency oscillations may improve mixing, but they also increase energy consumption. There is no mention of whether the energy cost associated with high oscillation frequencies offsets the gain in biodiesel yield.

Response: Thank you for your benefit comment. we have added a discussion in section (3.9) addressing the practical limitations of high-frequency oscillations. While increased frequencies enhance mixing and biodiesel yield, the energy consumption also increased duo to the greater oscillatory power requirements. Although specific energy costs were not quantified in this study, the significant yield improvement suggests a potential efficiency gain, warranting further investigation into energy trade-offs for industrial feasibility.

3. The manuscript models the reactor using a single-phase flow assumption with the k-ε turbulence model (Section 2.3.1). While k-ε is widely used, it struggles with near-wall turbulence predictions and may underpredict the effects of flow separation around baffles.
   The authors should briefly explain why they did not use a k-ω SST model which would provide a more accurate representation of turbulence and vortex shedding.

Response: Thanks for mentioning that. It was added in section (2.3.1). The main reason for choosing the k- ε model for CFD simulation is its efficiency and reliability in simulating the intense turbulent flows of an oscillatory reactor. It excels at capturing TKE and its dissipation rate, crucial for enhancing mixing in biodiesel production. While models like k-ω SST offer precision near walls, k-ε strikes an effective balance between accuracy and computational simplicity, ideal for focus on bulk flow dynamics.

4. The experimental validation section (2.5) states that biodiesel yield was measured using Gas Chromatography (GC), but does not provide details such as: GC sample preparation? The general settings of the GC? Specifications of the GC used? This will help with the reproducibility and comparison of results.

Response: Thanks for mentioning that. It was added in section (2.5.1).

5. I could not correlate the figure discussions to figures as the figures were not attached to the manuscript.

Response: Thanks for noting this issue. The figures were submitted in a separate file labeled "Figures" with the original manuscript.

Reviewer #3

1. Design the Abstract part, and it must be more informative by including more mathematical findings and more powerful explanations (For e.g., also discuss the parameters effect on biodiesel yield). Please be concise—maximum 1-2 lines.

Response: Thanks for your attention. corrected in the text.

2. The author should use new and more references in the introduction (2024, 2023, 2022, …).

Response: Thanks for your suggestion. corrected in the text.

3. For consistency, write the abbreviation of the terminology and use these abbreviations throughout the manuscript to avoid repetition of terminologies [e.g., turbulent kinetic energy (TKE), computational fluid dynamics (CFD), etc…].

Response: Thanks for your suggestion. It was added between abstract and introduction section.

4. In between the words, the capital words have been used. Please revise these.

Response: Thanks for your attention. It was corrected in the text.

5. In the result and discussion section, the authors discuss the result but mention a few outdated references. Please discuss your results with previous recent studies.

Response: Thanks for your attention. It was added in results and discussion section.

6. Can you explain the statistical procedures used to ensure reliable and accurate findings in the RSM modeling?

Response: Thank you for your question. Explanation was added to the different part of section (2). As it shown in section (2) ranges for d₀/D, frequency, and baffle spacing (Table 2) were derived, then used in RSM to generate 29 runs from a Box-Behnken design for reactor designs then simulating them with using CFD methods to obtain the outputs (TKE, dissipations, and vorticity), and then start optimizing based on outputs. The optimal condition simulated using CFD and the outputs compare with the optimal point of RSM. By comparing these two we understand that the optimal condition matched CFD results and experimental yield (83%), confirming robustness.

7. Citations are included but could be better integrated into the narrative. Some sections might benefit from additional references to support claims or provide further reading.

Response: Thanks for your attention. corrected in the text.

8. The author should consider adding references from the past few years (2020 onward) to reflect the latest developments in the field.

Response: Thanks for your attention. It was done.

9. In Table 1, author, please check the values of “the ratio of baffle diameter to reactor diameter” (For e.g., it is 0.4-0.8 or 0/4-0/8)?

Response: Thanks for mentioning that. It was corrected in the text.

10. Please check all the references cited in the text carefully and correct the inconsistency (For e.g., Line 554, check the reference format, i.e., Sreenivasan & Vedantam, 2024).

Response: Thanks for your attention. It was corrected in the text.

11. A professional proofread should be conducted again for the whole manuscript due to many typos.

Response: Thanks for your attention. It was revised.
