# Supplementary material for: Optimization and CFD-RSM analysis of single orifice reactor for enhanced biodiesel production
Source: PLoS One. 2025 Jul 2;20(7):e0326747. doi: 10.1371/journal.pone.0326747 (PMC12221185; doi:10.1371/journal.pone.0326747)
Supplement: Attachment — Submitted filename: PLOS ONE-R1 .docx [file pone.0326747.s005.docx]

**Paper Title**: Optimization and CFD-RSM Analysis of Single Orifice Reactor for Enhanced Biodiesel Production

**Journal Title:** PLOS ONE

**Manuscript No.:** PONE-D-25-06866R1

**Article type:** Research Article

In the present investigation titled “Optimization and CFD-RSM Analysis of Single Orifice Reactor for Enhanced Biodiesel Production” demonstrates the potential of single orifice reactor to enhance biodiesel production through improved turbulance and mixing efficiency. However, after reviewed the revised manuscript carefully, the authors have successfully resolved the minor queries, so I recommend the manuscript for the publication.
