# Supplementary material for: Optimization and CFD-RSM analysis of single orifice reactor for enhanced biodiesel production
Source: PLoS One. 2025 Jul 2;20(7):e0326747. doi: 10.1371/journal.pone.0326747 (PMC12221185; doi:10.1371/journal.pone.0326747)
Supplement: Attachment — Submitted filename: response.docx [file pone.0326747.s007.docx]

Response to the honorable referee of the paper entitled:

**Optimization and CFD-RSM Analysis of Single Orifice Reactor for Enhanced Biodiesel Production**

Thanking the comments and proposed amendments of the honorable referees, the answer to the referred points is mentioned in the separation of different parts of the paper as follows:

Reviewer #1

1. The manuscript has improved in only a few sections and requires deeper corrections before publication. Re-check all previous comments and submit a highlights version, specifying the accurate location of changes in the revised version.

Response: Thanks for your feedback. All previous comments are checked and the changes are added in the revised manuscript.

2. The methods section is not adequately explained. Provide a more detailed description of the experimental setup, including reactor dimensions, operating conditions, and chemical reagent purity.

Response: Thanks for your attention. It was corrected in text.

3. The parameter of enhancement rate does not add value to the discussion and should be reconsidered or removed.

Response: Thanks for your recommendation. It was removed.

4. The discussion of the results is vague and needs to be more detailed. Include a comparison with recent studies on similar reactor designs or biodiesel production processes to better position the findings.

Response: Thanks for pointing that out. It was corrected in text.

5. Avoid using first-person pronouns ("we," "us," "our") throughout the paper to maintain a formal tone.

Response: Thanks for pointing that out. It was corrected in the text.

6. Redaction and English grammar need revision. Some sentences require clarification and strengthening to improve readability and professionalism.

Response: Thanks for your feedback. It was revised.

7. Remove lumped references and define each citation separately to avoid inappropriate citations.

Response: Thanks for your suggestion. It was corrected in text.

8. The unique contribution of the paper is unclear. Highlight this in the abstract, which currently narrates activities rather than addressing key points: what the study aims to do, why, what was found, and the significance of the findings.

Response: Thanks for your suggestion. The abstract of this paper was revised.

9. The introduction lacks a proper state-of-the-art overview. Review recent developments in the field (e.g., studies like "Development of Heterogeneous/Nanocatalysts in Biodiesel Production," "Magnetic graphene oxide supported tin oxide (SnO) nanocomposite as a heterogeneous catalyst for biodiesel production," "Exploring Waste-Derived Catalysts for Sustainable Biodiesel Production") to support the research gap and significance. Improve the aim of the introduction to show progress beyond the state of the art.

Response: Thank you for your valuable suggestion. The references have been incorporated in lines (102-107).

10. Provide quantitative comparisons between CFD predictions and experimental results (e.g., biodiesel yield, reaction conversion rates) with statistical analysis to validate the simulation approach.

Response: Thanks for your suggestion. we have added a detailed comparison in Section 3.8, lines (773-783).

11. Clarify how the optimized conditions (frequency of 12.12 Hz, d₀/D of 0.4, baffle spacing of 10 mm) were selected from the tested ranges. Discuss trade-offs between turbulence metrics (TKE, vorticity) and biodiesel yield, supported by data or graphs.

Response: Thanks for your comment. A flowchart was added to section (2) to clarify the process. And also, some explanations were added to section (3.8). as it shown ranges for d₀/D, frequency, and baffle spacing (Table 2) were derived from Section (2.2) equations, then used in RSM to generate different options for reactor designs. These were simulated using CFD methods to obtain TKE, dissipations, and vorticity, which RSM analyzed to find the optimal point. Experiments validated this with an 83% biodiesel yield, linked to improved mixing. Among the tested ranges, these conditions outperformed others by balancing high TKE and vorticity with lower energy dissipation leading to the highest biodiesel yield. Also, we have revised Section 3.8 (Process Optimization) to include a detailed explanation of how the specific values were determined using the Response Surface Method (RSM) and the trade-offs considered.

12. Specify the turbulence model used (k-ε, k-ω, LES) in the CFD simulations, with justification for its selection. Explain how mesh independence and boundary conditions were determined to ensure simulation accuracy.

Response: Thanks for your suggestion. The manuscript already specifies the use of the k-ε turbulence model in Section 2.3.1 (K-ε Turbulent Model) in lines (205-215), but we have expanded this section to include a more detailed justification for choosing the k-ε model over other models such as k-ω or Large Eddy Simulation (LES).

The importance of discussing mesh independence and boundary conditions were acknowledged to ensure the reliability of our CFD simulations. To address this, new subsection was added in Section 2.3 (in lines 180-197) to detail the mesh independence study and boundary condition determination.

13. The conclusion is generic and does not contribute to the existing knowledge base. Revise it to be concise, emphasizing the scientific value, novelty, practical implications (e.g., scalability, environmental benefits, integration into biodiesel production workflows), and quantitative benchmarks or comparisons.

Response: Thanks for your suggestion. The conclusion section was revised.

14. Ensure all references are appropriately cited in the main text and that figure captions are consistently formatted.

Response: Thanks for your attention. The references have been checked, additionally, captions of figures (1) and (10) were corrected in the manuscript and also in the attachment file.
